# The clinical effect of probiotics on patients with non-alcoholic fatty liver disease: a meta-analysis

**DOI:** 10.1080/21655979.2023.2185941

**Published:** 2023-04-27

**Authors:** Yuxue Wang, Yarong Wang, Jianguang Sun

**Affiliations:** a Department of hepatology, The first clinical medical college of Shandong University of traditional Chinese Medicine Jinan, China; b Department of Internal medicine of traditional Chinese Medicine, Jinan Shi Minzu Hospital

**Keywords:** Nonalcoholic fatty liver disease (NAFLD), probiotics, clinical effect, meta-analysis, TNF-α

## Abstract

Nonalcoholic fatty liver disease (NAFLD) is a common chronic liver disease. The present study explores the clinical efficacy of probiotics in the treatment of patients with NAFLD by conducting a systematic search of relevant databases. The RevMan 5.4 software was used to evaluate the effects of probiotics on liver function (i.e. alanine aminotransferase [ALT], aspartate aminotransferase [AST], gamma-glutamyl transferase [GGT], lipid metabolism, blood glucose, inflammatory factors [e.g. tumor necrosis factor-α, TNF-α] and body mass index [BMI]) in patients with NAFLD. A total of 18 high-quality studies were included in the final meta-analysis. The results of the meta-analysis showed that the use of probiotics in the adjuvant treatment of patients with NAFLD improved liver function and reduced ALT levels (mean difference [MD]: −0.07; 95% confidence interval [CI]: −12.95, −7.19), AST levels (MD: −11.90; 95% CI: −16.55, −7.25) and GGT levels (MD: −8.61; 95% CI: −14.74, −2.48); additionally, the treatment effect was more obvious when the treatment time exceeded 12 weeks. Probiotic therapy reduced patients’ triglyceride levels (MD: −9.71; 95% CI: −18.39, −1.03) and total cholesterol levels (MD: −22.31; 95% CI: −25.41, −19.21). Probiotic treatment improved patients’ levels of fasting blood (MD: −8.22; 95% CI: −12.25, −4.20), insulin (MD: −2.68; 95% CI: −4.94, −0.41) and insulin resistance (MD: −0.72; 95% CI: −1.21, −0.24). Probiotic adjuvant therapy for patients with NAFLD reduced their BMI by approximately 1.67 (95% CI: −2.93, −0.41) and TNF-α levels. The adjuvant treatment of NAFLD with probiotics has a positive clinical effect, which is influenced by treatment time.

## GRAPHICAL ABSTRACT

**Figure:**
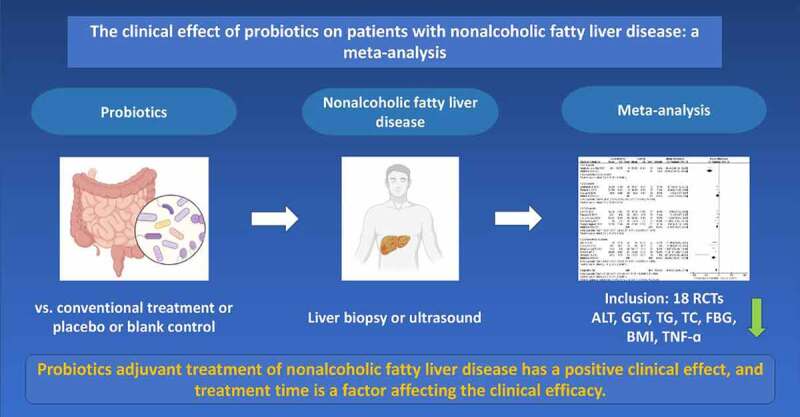

## 1 Introduction

Nonalcoholic fatty liver disease (NAFLD) is an acquired stress liver disease caused by excessive fat deposition in hepatocytes in addition to injury induced by alcohol and other causes [1]. With the global improvement in living standards and the prevalence of obesity and metabolic syndrome, NAFLD has become a common chronic liver disease across the world and has attracted wide attention from all circles of society. According to a meta-analysis of global NAFLD in 22 countries, the prevalence rate of global NAFLD is 25.2%, and the mortality rate is 11.77% [2]. In addition, studies on NAFLD in China showed that its prevalence in China is not optimistic. Large-sample epidemiological survey results revealed that the prevalence of NAFLD in China is higher than in Europe and the United States; it surged from 25.4% in 2008–2010 to 32.3% in 2015–2018 [3]. Thus, without timely and effective treatment, NAFLD will become a major threat to the health of global citizens.

However, the exact pathogenesis of NAFLD has not been fully elucidated, and there is a lack of effective prevention and treatment measures. A study revealed that patients with NAFLD had an increased abundance of *Anaerococcus* (Actinobacteria), *Ruminococcus* (Firmicutes), *Peptoniphilus* (Firmicutes), *Dorea* (Firmicutes), *Bradyrhizobium* (Proteobacteria) and *Propionibacterium acnes* (Actinobacteria) but a reduced abundance of *Rikenellaceae* (Bacteroidetes) and *Oscillospira* (Firmicutes) [4]. In addition, studies have shown that intestinal flora imbalance of various causes, including dietary factors, can lead to decreased intestinal mucosal barrier function and increased inflammatory factors, thereby affecting liver metabolism and function via the gut – liver axis and promoting the occurrence and development of NAFLD [5,6]. As active microorganisms, probiotics can improve the intestinal microbial system and promote healthy outcomes in the host when they are used in sufficient quantities [7]. A large number of animal experiments have found that an intake of probiotics (e.g. *Lactobacillus, Bifidobacterium, Streptococcus, Enterococcus, Bacillus and Saccharomyces*) can improve liver steatosis in animals with NAFLD and promote normal liver function, indicating that probiotics can prevent the occurrence and development of NAFLD by maintaining the balance of intestinal flora [8–10]. In recent years, several clinical trials have explored the effects of probiotics on liver function, blood glucose and lipid metabolism in patients with NAFLD, but the conclusions remain controversial.

Therefore, this study aimed to systematically retrieve relevant Chinese and English databases and evaluate the clinical effect of probiotics in the adjuvant treatment of patients with NAFLD via a meta-analysis to provide a scientific theoretical basis for the use of probiotics in the treatment of NAFLD.

## 2 Materials and methods

### 2.1 Search strategy

Following the PRISMA guidebook [11], a systematic literature search of the PubMed, Embase, Cochrane Library, Web of Science, Cumulative Index to Nursing and Allied Health Literature, China National Knowledge Infrastructure, Wanfang and VIP databases was performed from the date of inception of the databases to 31 March 2022. A search strategy combining subject headings and free words was used. The search terms included ‘nonalcoholic fatty liver disease,’ ‘NAFLD,’ ‘nonalcoholic steatohepatitis,’ ‘probiotics’ and ‘*Lactobacillus*.’ The term ‘randomized controlled trials’ (RCTs) and RCT synonyms of each term were also used. The target literature was obtained by reading the relevant systematic review.

### 2.1 Inclusion and exclusion criteria

The inclusion criteria were as follows: (1) patients with NAFLD who were examined by liver biopsy or ultrasound; (2) patients in the experimental group who underwent probiotic treatment without dose limitation; (3) patients in the control group who received conventional treatment with non-probiotics, a placebo or a blank control; (4) patients with outcome indicators including alanine aminotransferase (ALT), aspartate aminotransferase (AST), gamma-glutamyl transferase (GGT), low-density lipoprotein (LDL) cholesterol, triglyceride (TG), fasting blood glucose, insulin, insulin resistance, body mass index (BMI) and tumor necrosis factor-α (TNF-α); and (5) only patients who were in RCTs.

The exclusion criteria were as follows: (1) patients with hepatic steatosis or hepatic fibrosis caused by viral hepatitis, autoimmune hepatitis, drug hepatitis or genetics; (2) non-demographic studies; (3) conference articles, case reports, systematic reviews and other research types; (4) studies with insufficient outcome information that could not be analyzed; (5) repeated reports of literature research; and (6) incomplete research articles.

### 2.3 Study selection and data extraction

Two reviewers independently reviewed the abstracts and full text of each article according to the inclusion and exclusion criteria. In the case of discrepancies between the two reviewers, a third reviewer was recruited for discussion until a consensus was achieved. After the literature screening, two reviewers independently extracted the following information: first author, publication time, country, demographic characteristics of subjects, probiotic treatment scheme and the mean and standard deviation of the outcome indicators. If only the median and range were reported in the literature, the data were further converted into the form of mean and standard deviation [12].

### 2.4 Assessment of methodological quality

The Cochrane Collaboration risk assessment tool [13] was used to evaluate the quality of the literature. The method was evaluated from the perspectives of the random distribution method, allocation concealment, the blind method, integrity of the results data, selective reporting of research results and other sources of bias.

### 2.5 Statistical analysis

The RevMan 5.3 software was used for the statistical analysis. The effect of the numeration data and the measurement data were expressed using the mean difference (MD), and the 95% confidence interval (CI) was used to estimate the interval range of the effect. A heterogeneity test was used to determine the degree of heterogeneity according to $I^{2}$ [14]. If $I^{2}$<50% or *p* > 0.1, the included literature was considered homogeneous, and the fixed-effect model (Mantel – Haenszel) was used for analysis; if $I^{2}$>50% or *p* ≤ 0.1, the included studies were considered heterogeneous, and the random-effect model (Der Simonian – Laird) was used for analysis. If the heterogeneity was large, subgroup and sensitivity analyses were used to explore the source of the heterogeneity. A value of *p* < 0.05 indicated that the difference was statistically significant.

## 3 Results

Nonalcoholic fatty liver disease is an increasing threat to human health. This study aimed to explore the clinical efficacy of probiotics in the treatment of NAFLD through a meta-analysis. Based on the systematic analysis of 18 pieces of literature that met the criteria of this study, the results showed that the use of probiotics in the adjuvant treatment of patients with NAFLD can improve the levels of fasting blood glucose and insulin, along with insulin resistance and liver function (i.e. it can reduce the levels of ALT, AST and GGT). In addition, probiotic treatment can reduce patients’ TG, total cholesterol and TNF-α levels and lower their BMI. The longer the treatment time, the more significant the effect.

### 3.1 Study characteristics

**Figure 1.**
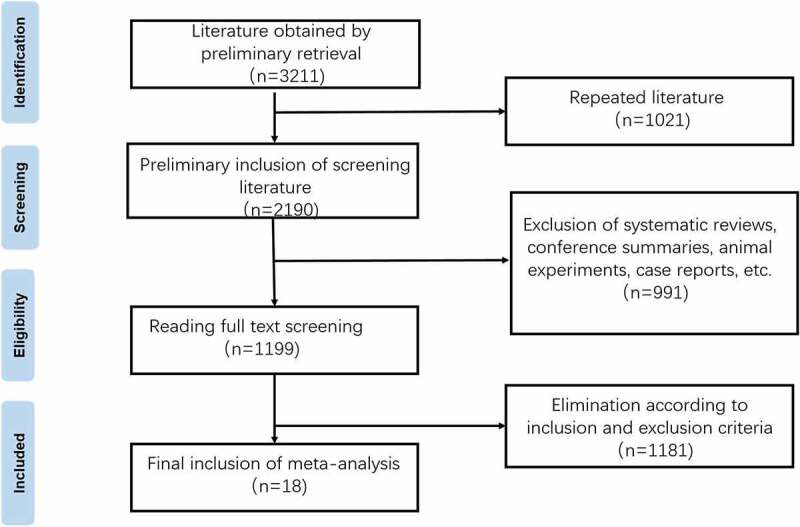
Flow chart of literature selection.

After systematic retrieval and screening, 18 pieces of literature met the present study’s inclusion and exclusion criteria [15–32]. The flow chart for literature retrieval and screening is shown in Figure 1. Eighteen studies involved 1,136 patients with NAFLD. Ultrasound examination was the most commonly used diagnostic criterion. A total of 14 studies used ultrasound examination, and liver biopsy was used for diagnosis. The research subjects of two studies were children, and the other research subjects were adults. The probiotics mainly included *Lactobacillus*, *Bifidobacterium*, *Streptococcus* and *Enterococcus*. The shortest treatment time was 1 month, and the longest treatment time was 1 year. Detailed basic features of the included studies are shown in Table 1.

**Table 1.** Inclusion of the basic features of the study.

study	country	definite diagnosis	number(intervention/control)	age(intervention/control,y)	female(intervention/control,%)	BMI(intervention/control,kg/m2)	Probiotic components	intervention time
Ferolla SM,2016	Brazil	hepatic biopsy	26/23	57.3(25–74)	38(76)	32.5 ± 4.0/32.5 ± 4.0	lactobacillus	12 weeks
Bakhshimoghaddam F,2018	Iran	ultrasonic examination	34/34	38.8 ± 9.0/41.1 ± 8.5	17(50)/17(50)	30.5 ± 4.6/31.9 ± 5.1	bifidobacterium, lactobacillus	24 weeks
Ekhlasi G,2016	Iran	ultrasonic examination	15/15	25–64	NR	27.28 ± 2.21/27.84 ± 1.96	lactobacillus, bifidobacterium, streptococcus	8 weeks
Sepideh A,2015	Iran	ultrasonic examination	21/21	42.10 ± 1.99/47.33 ± 2.53	8(38.1)/6(28.6)	30.34 ± 1.17/29.50 ± 0.84	lactobacillus, bifidobacterium, streptococcus	8 weeks
Behrouz V,2017	Iran	ultrasonic examination	30/30	38.46 ± 7.11/38.43 ± 10.09	8(26.7)/9(30)	29.56 ± 2.54/31.90 ± 5.04	lactobacillus, bifidobacterium	12 weeks
Famouri F,2017	Iran	ultrasonic examination	32/32	12.7 ± 2.2/12.6 ± 1.7	18(56.2)/14(43.8)	26.44 ± 4.3/26.61 ± 2.26	lactobacillus, bifidobacterium	12 weeks
Duseja A,2019	India	hepatic biopsy	19/20	38 ± 10/33 ± 6	6(32)/5(25)	26 ± 3/27 ± 4	lactobacillus, bifidobacterium	12 months
Abdel Monem SM,2017	Egypt	hepatic biopsy	15/15	44.20 ± 5.51/44.33 ± 5.62	6(40)/7(46.7)	32.56 ± 1.19/33.05 ± 1.27	lactobacillus	1 months
Cai GS,2020	China	ultrasonic examination	70/70	46.13 ± 12.72/49.62 ± 9.08	24(34.29)/31(44.29)	31.28 ± 3.62/30.73 ± 3.47	lactobacillus, bifidobacterium, enterococcus	3 months
Alisi A,2014	Italy	ultrasonic examination	22/22	11(10–12)/10(9–12)	8(36.36)/12(54.55)	25.6(23.2–27.9)/27.3(24.7–28.6)	lactobacillus, bifidobacterium, streptococcus	16 weeks
Mofidi F,2017	Iran	ultrasonic examination	21/21	40.09 ± 11.44/44.61 ± 10.12	10(47.62)/9(42.86)	23.17 ± 1.01/23.20 ± 1.07	lactobacillus, bifidobacterium, streptococcus	28 weeks
Eslamparast T,2014	Iran	ultrasonic examination	26/26	46.35 ± 8.8/45.69 ± 9.5	12(46.15)/15(57.69)	32.1 ± 2.4/31.3 ± 2.3	lactobacillus, bifidobacterium, streptococcus	28 weeks
Jiang Yanyan,2020	China	ultrasonic examination	36/36	51.84 ± 10.46/51.56 ± 14.83	14(38.89)/16(44.44)	NR	bifidobacterium	12 weeks
Wang Lingjuan,2019	China	ultrasonic examination	60/60	6–14	NR	NR	bifidobacterium	3 months
Shavakhi A,2013	Iran	hepatic biopsy	32/31	41.5 ± 12.7/38.7 ± 11.9	17(53.13)/14(45.16)	28.6 ± 2/28.2 ± 2.5	lactobacillus, bifidobacterium, streptococcus	6 months
Nabavi S,2014	Iran	ultrasonic examination	36/36	42.75 ± 8.72/44.05 ± 8.14	19(52.8)/18(50)	30.1 ± 3.61/31.4 ± 3.6	lactobacillus, bifidobacterium	8 weeks
Asgharian A,2016	Iran	ultrasonic examination	38/36	46.57 ± 1.7/47.78 ± 1.7	33(82.5)/22(64.7)	29.58 ± 0.76/28.18 ± 0.68	lactobacillus, bifidobacterium, streptococcus	8 weeks
Manzhalii E,2017	Ukraine	ultrasonic examination	38/37	44.3 ± 1.5/43.5 ± 1.3	27(71.05)/21(56.76)	26.4 ± 0.8/26.6 ± 0.7	lactobacillus, bifidobacterium, streptococcus	12 weeks

### 3.2 Literature quality evaluation

**Figure 2.**
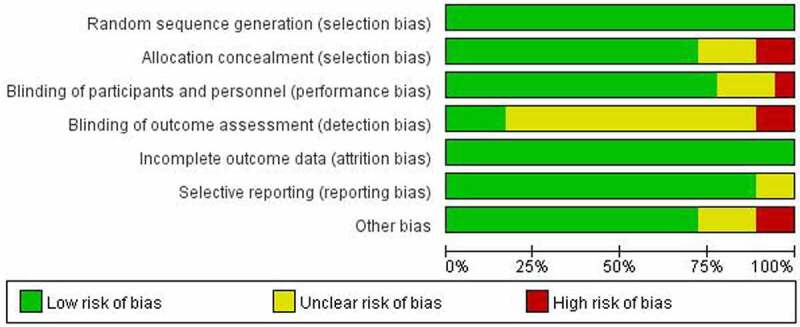
Quality evaluation results of included studies.

**Figure 3.**
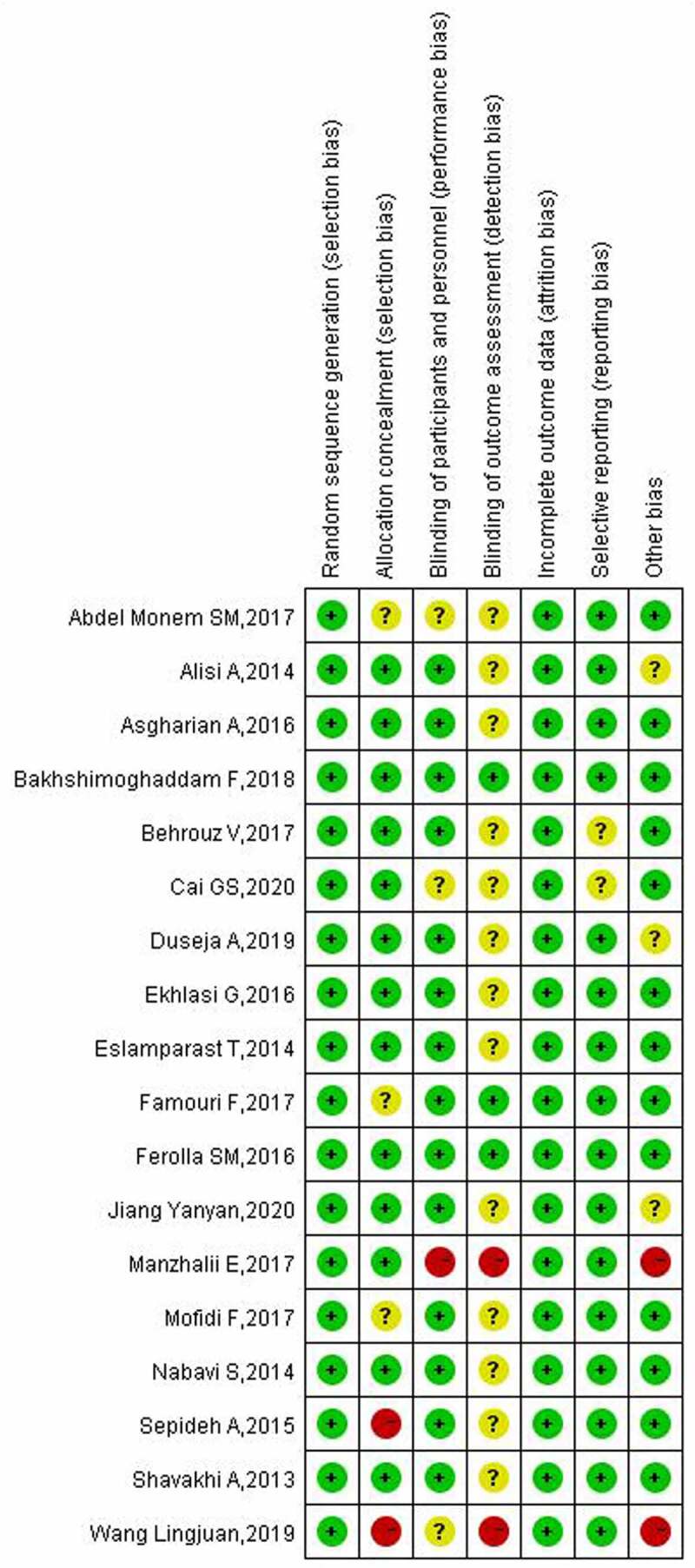
End map of risk of bias included in the study.

The results of the literature quality evaluation showed that the 18 studies included were of high quality, and the generation and outcome integrity of random sequences were the two aspects with the lowest bias risk. However, there was a certain risk of bias in the implementation of the blind law. The results of the literature quality evaluation are shown in Figures 2 and 3.

### 3.3 Effect of probiotics on liver function in patients with NAFLD

**Figure 4.**
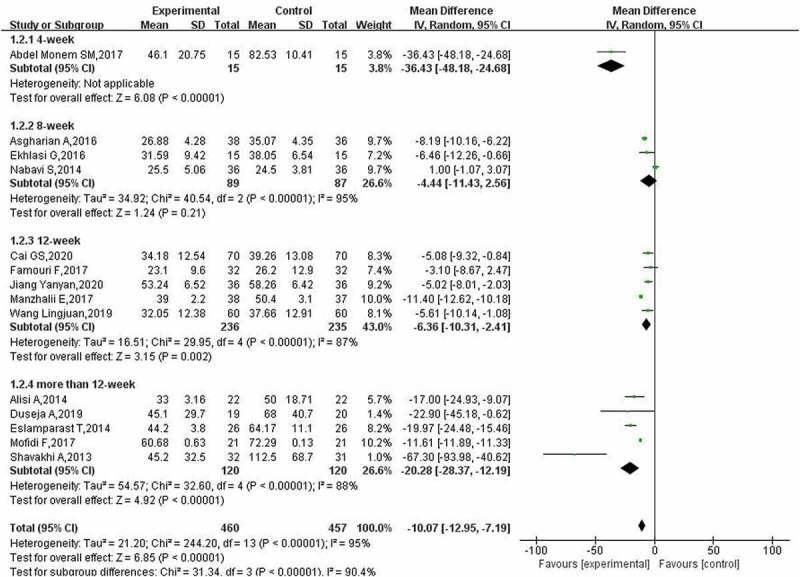
Effect of probiotics on ALT (IU/L) in NAFLD patients.

In 14, 12 and 5 studies, the results of liver function indexes (regarding ALT, AST and GGT, respectively) in patients with NAFLD after using probiotics were reported. The heterogeneity analysis of ALT indicators showed that the heterogeneity among the included studies was high (*p* < 0.00001), and the random-effect model was used for analysis. The subgroup analysis results (Figure 4) showed that the use of probiotics in the adjuvant treatment of NAFLD reduced ALT levels (MD: −10.07; 95% CI: −12. 95, −7. 19), and the effect with more than 12 weeks of treatment was more obvious (MD: − 20. 28; 95% CI: −28.37, −12.19). However, the present study did not find that 8 weeks of treatment had a positive effect on ALT levels (MD: −4.44; 95% CI: −11.43, 2.56).

**Figure 5.**
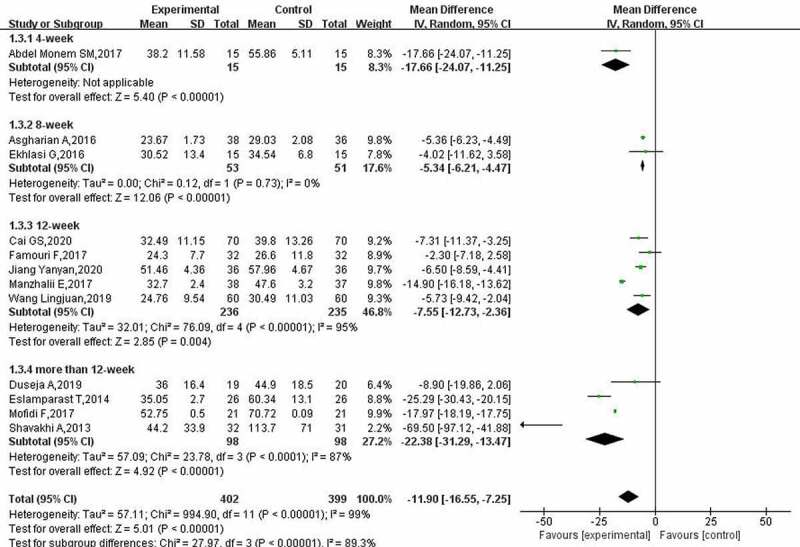
Effect of probiotics on AST (IU/L) in NAFLD patients.

The results of the heterogeneity analysis of AST indicators showed that there was a high degree of heterogeneity among the included studies (*p* < 0.00001). The random-effect model was used, and a subgroup analysis was performed. The meta-analysis results for AST (Figure 5) showed that the use of probiotics in patients with NAFLD could reduce AST levels (MD: −11.90; 95% CI: −16.55, −7.25). The subgroup analysis revealed that except for only one treatment time of 4 weeks, the reduction in AST was related to the treatment time. When the treatment time exceeded 12 weeks, the AST levels of patients decreased by 22.38 (95% CI: −31.29, −13.47).

**Figure 6.**
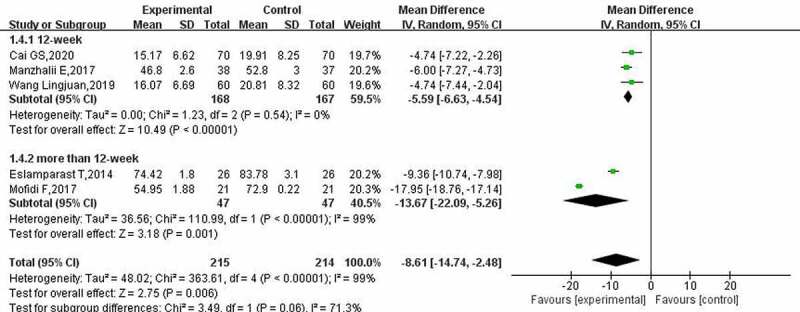
Effect of probiotics on GGT (IU/L) in NAFLD patients.

According to the heterogeneity test results of the GGT index (I^2^ = 99%, *p* < 0.00001), the random-effect model was used for analysis. The results of the meta-analysis of GGT (Figure 6) showed that probiotics could reduce GGT levels in patients with NAFLD (MD: −8.61; 95% CI: −14.74, −2.48). In addition, the reduction in GGT after treatment for more than 12 weeks was significantly higher than the reduction after treatment for 12 weeks.

### 3.4 Effect of probiotics on lipid metabolism in patients with NAFLD

**Figure 7.**
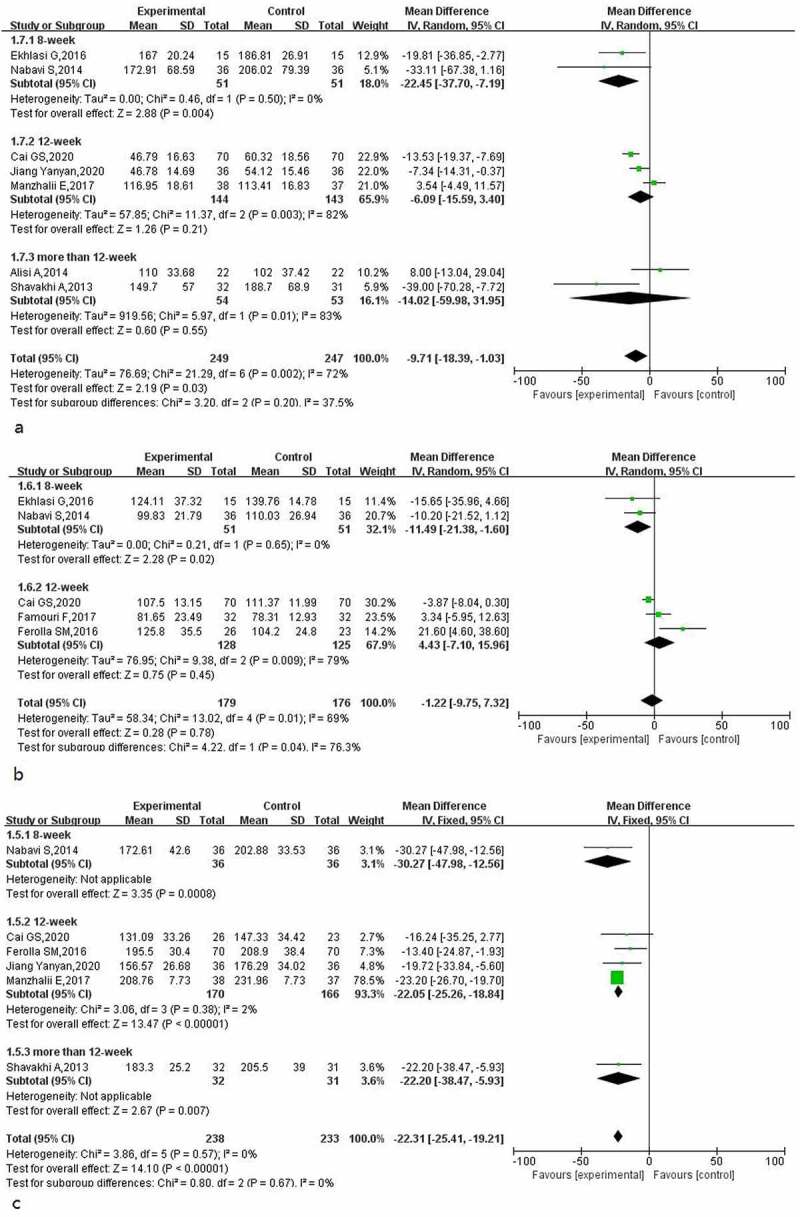
Effect of probiotics on triglyceride (mg/L) in NAFLD patients; b: Effect of probiotics on low-density protein cholesterol (mg/L) in patients with NAFLD; c: Effect of probiotics on total cholesterol (mg/L) in NAFLD patients.

A total of 7, 5 and 6 studies reported on the changes in lipid metabolism (regarding TG, LDL cholesterol and total cholesterol, respectively) in patients with NAFLD after probiotic treatment. The heterogeneity test results for TG (I^2^ = 72%, *p* = 0.002) indicated that the homogeneity of the included studies was poor. The results were analyzed by the random-effect model. The subgroup analysis results for TG (Figure 7 a) showed that probiotic treatment could reduce patients’ TG levels (MD: −9.71; 95% CI: −18. 39, −1. 03); however, there were some differences between treatment times. The effect of a treatment time of 12 weeks and above on TG levels in patients with NAFLD was not statistically significant (12 WKS, *p* = 0.21; >12 WKS, *p* = 0.55).

The heterogeneity test results for LDL cholesterol showed that there was a certain heterogeneity between the included studies (I^2^ = 69%, *p* = 0.01), and the random-effect model was used to calculate the combined effect. The meta-analysis results for LDL cholesterol (Figure 7 a) showed that probiotic treatment did not improve LDL cholesterol levels in patients (MD: −1.22; 95% CI: −9.75, 7.32); however, when the treatment time was 8 weeks, probiotic treatment reduced LDL cholesterol (MD: −11.49; 95% CI: −21.38, −1.60).

The heterogeneity evaluation results of total cholesterol suggested that the included studies had good homogeneity (I^2^ = 0%, *p* = 0.57), and the fixed-effect model was used for evaluation. The meta-analysis of total cholesterol (Figure 7c) showed that after probiotic treatment, the total cholesterol level of patients with NAFLD decreased by−22.31 (95% CI: −25.41, −19.21), and different treatment times had the same effect.

### 3.5 Effect of probiotics on blood glucose in patients with NAFLD

**Figure 8.**
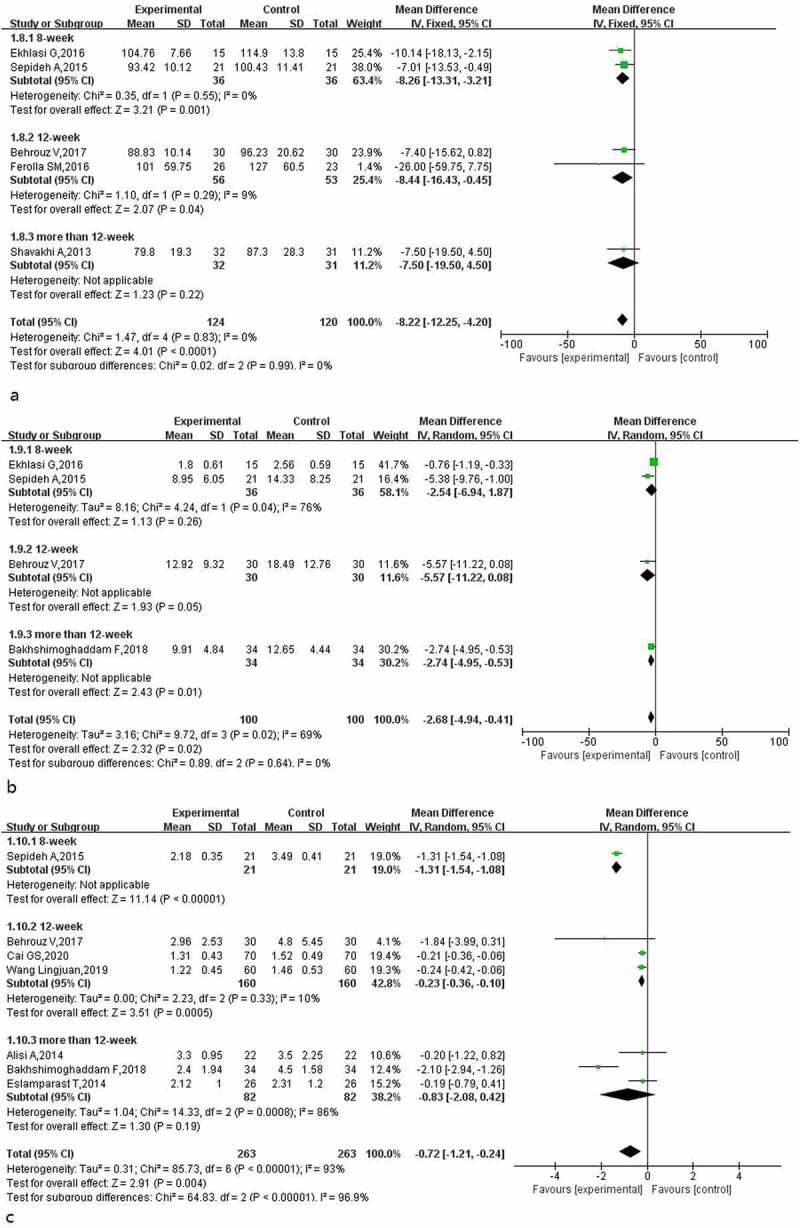
Effect of probiotics on fasting blood glucose (mg/L) in NAFLD patients; b: Effect of probiotics on insulin (IU/L) in NAFLD patients; c: Effect of probiotics on insulin resistance in NAFLD patients.

A total of 5, 4 and 7 studies reported the effects of probiotics on blood glucose (regarding fasting blood glucose, insulin and insulin resistance, respectively) in patients with NAFLD. The heterogeneity evaluation results for fasting blood glucose (I^2^ = 0%, *p* = 0.83) showed that the heterogeneity among the included studies was low, and the fixed-effect model was used for evaluation. The results of the fasting blood glucose meta-analysis (Figure 8a) indicated that probiotic treatment improved the fasting blood glucose level in patients (MD: −8.22; 95% CI: −12. 25, −4.20).

The heterogeneity test results for insulin indicators (I^2^ = 69%, *p* = 0.02) suggested that there was certain heterogeneity in the included studies, and the random-effect model was used for evaluation. The insulin meta-analysis results (Figure 8b) suggested that probiotic treatment reduced insulin levels in patients (MD: −2.68; 95% CI: −4.94, −0.41), but when the treatment times were 8 and 12 weeks, the improvement effect of probiotics on insulin in patients was not statistically significant.

The heterogeneity test results for insulin resistance indicators (I^2^ = 93%, *p* = 0.0008) suggested that the heterogeneity among the included studies was high, and the random-effect model was used to calculate the combined effect. The subgroup analysis results for insulin resistance (Figure 8c) showed that heterogeneity was mainly derived from three studies with treatment times exceeding 12 weeks, and probiotic treatment had a positive effect on improving insulin resistance in patients (MD: −0.72; 95% CI: −1.21, −0.24); however, when the treatment time exceeded 12 weeks, the improvement effect of probiotics on insulin resistance was not statistically significant (MD: −0.83; 95% CI: −2.08, 0.42).

### 3.6 Effect of probiotics on BMI and inflammatory factors in patients with NAFLD

**Figure 9.**
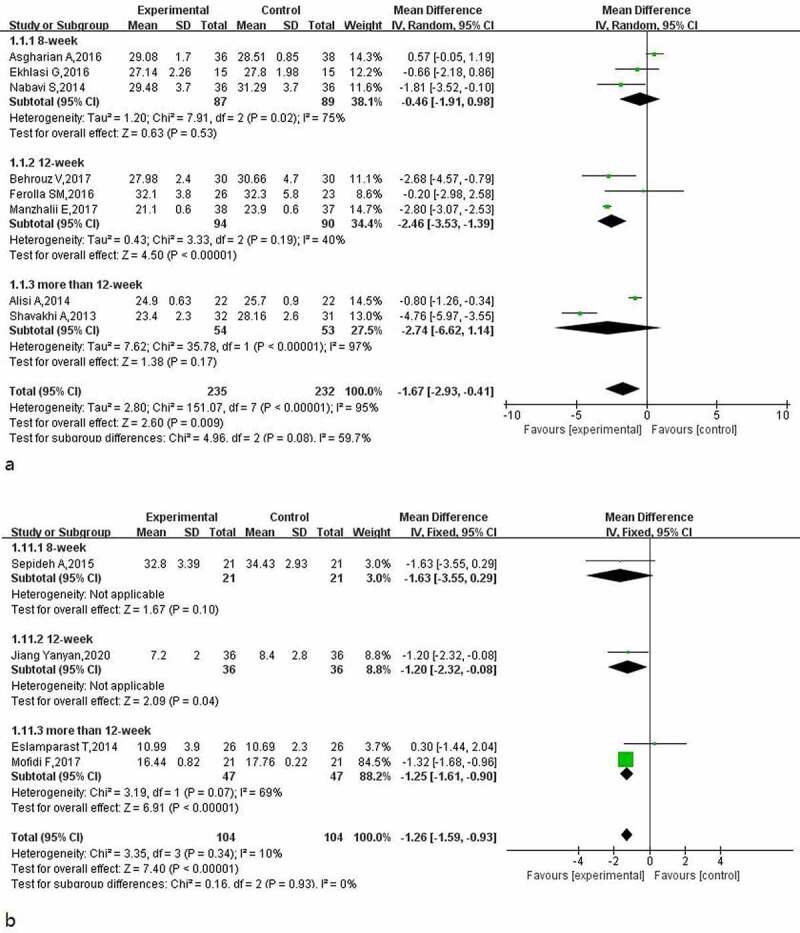
Effect of probiotics on BMI (Kg/$m^{2}$) in NAFLD patient; b: Effect of probiotics on TNF-α (pg/ml) in NAFLD patients.

The results for BMI and TNF-α in patients with NAFLD after using probiotics were reported in 8 and 4 studies, respectively. The heterogeneity test results for BMI (I^2^ = 95%, *p* < 0.00001) meant that the random-effect model was used to calculate the combined effect. The results of the BMI subgroup analysis (Figure 9a) showed that the use of probiotics in the adjuvant treatment of patients with NAFLD reduced the BMI of patients by about 1.67 (95% CI: −2.93, −0.41), and the effect of different treatment times was significantly different. When the treatment time was 12 weeks, the positive effect of probiotics on BMI was statistically significant.

The heterogeneity test results for inflammatory factor TNF-α (I^2^ = 10%, *p* = 0.34) were evaluated using the fixed-effect model. The meta-analysis results for TNF-α (Figure 9b) showed that when the treatment time was 12 weeks or longer, probiotic treatment reduced the TNF-α level in patients. The longer the treatment time, the more obvious the effect. The total combined effect was−1.26 (95% CI: −1.59, −0.93).

## 4 Discussion

In the present study, 18 high-quality RCTs were included in a meta-analysis. The components of the probiotics mainly included *Lactobacillus*, *Bifidobacterium*, *Streptococcus* and *Enterococcus*. The treatment duration ranged from 1 month to 1 year. We focused on the effects of probiotics on liver function, lipid metabolism, blood glucose, BMI and inflammatory factors in patients with NAFLD. The results showed that probiotic treatment had significantly positive effects on ALT, AST, GGT, TG, total cholesterol, fasting blood glucose, insulin, insulin resistance, BMI and TNF-α in patients with NAFLD; however, the positive effect of probiotics on LDL cholesterol was not statistically significant. In addition, the study found that treatment duration had a significant effect on clinical efficacy. Subgroup analyses were performed according to treatment times of 4, 8, 12 and 12+ weeks. The subgroup analysis results showed that when the treatment time was longer than 12 weeks, the improvement in ALT, AST and GGT levels in patients with NAFLD was more significant. However, when the treatment time exceeded 12 weeks, the improvement effect of probiotics on TG, fasting blood glucose and insulin resistance was not statistically significant.

The mechanism between the duration of treatment and clinical outcome is still unclear; however, the underlying relationship may be that prolonged probiotic treatment is more beneficial for the recovery of gut microbiota in patients with NAFLD [33].

Previous studies have shown that probiotics have significant therapeutic effects on patients with NAFLD. For instance, Liu et al. [34] showed that supplementing probiotics reduced AST levels in patients with NAFLD (MD: −13.11; 95% CI: −17.37, −8.85) as well as ALT levels (MD: −13. 95; 95% CI: −16.12, −11.78). Tang and Xie et al. [35,36] indicated that after probiotic treatment, the total cholesterol, LDL cholesterol and fasting blood glucose of patients with NAFLD decreased significantly.

However, it is worth noting that these studies did not further consider the impact of treatment time on the therapeutic effect. The results of the present study suggested that there were no obvious differences in therapeutic effect among different treatment times.

Probiotics can improve intestinal flora imbalance and regulate intestinal microbial metabolites. They are a widely accepted mechanism for improving NAFLD. A comparative study of intestinal microflora between patients with NAFLD and non-obese healthy people found that the number of gram-negative bacteria in patients with NAFLD was higher, with an increase in the number of Bacteroidetes by 20%; furthermore, similar changes in intestinal microbiota were also found in children [37].

Diet, obesity, infection, drug treatment and other factors affect intestinal flora, resulting in increased intestinal permeability, intestinal flora overgrowth, bacterial translocation and lipopolysaccharide release, thereby inducing the release of inflammatory cytokines and resulting in the occurrence of NAFLD [38,39]. Koutnikova et al. [40] evaluated the effect of oral probiotics on obesity, diabetes and NAFLD. The results showed that the intake of probiotics improved some metabolic factors in patients with metabolic diseases.

Furthermore, different probiotic types may have different effects on NAFLD. An animal model showed that two strains of *Lactobacillus rhamnosus* LGG and L10–1 relieved NAFLD in different ways; LGG modulated the energy metabolism and lipid metabolism, while L10–1 reduced liver inflammation. Strains of *Bifidobacterium adolescentis* relieved NAFLD by increasing the concentration of short-chain fatty acids in the intestine of NAFLD mice [41].

The present study has certain advantages and limitations. First, it comprehensively retrieved data from Chinese and English databases to determine the effect of probiotics on patients with NAFLD in China. Second, it fully considered the clinical effect of treatment duration on probiotic treatment through subgroup analyses of the treatment duration. However, due to the differences in research population and treatment regimens, there is a certain heterogeneity between the studies. In addition, because only one study treated subjects for 4 weeks, the evaluation results of the treatment time have certain limitations. Finally, four studies used liver biopsy to diagnose patients with NAFLD, and it is unknown whether this traumatic pathological diagnosis affected the therapeutic effect of probiotics.

## 5 Conclusions

In summary, supplementing probiotics, including *Lactobacillus*, *Bifidobacterium*, *Streptococcus* and *Enterococcus*, had a significantly positive effect on improving liver function, lipid metabolism, blood glucose, BMI and inflammatory factors in patients with NAFLD. Moreover, the clinical effects caused by different durations of probiotic treatment were significantly different. The present study suggests that supplementing probiotics is a recommended method for the treatment of patients with NAFLD. However, based on the limitations of this study, a large number of high-quality RCTs are needed in the future to explore the effect of treatment duration on the therapeutic efficacy of probiotics.

## Data Availability

All data generated or analyzed during this study are included in this published article.

## Highlights

1. Probiotic adjuvant therapy for NAFLD has positive clinical efficacy.
2. The treatment time is the factor affecting the clinical efficacy.
3. Probiotics in the adjuvant treatment of patients with NAFLD can improve liver function.
